# Biostatistics mining associated method identifies AKR1B10 enhancing hepatocellular carcinoma cell growth and degenerated by miR-383-5p

**DOI:** 10.1038/s41598-018-29271-3

**Published:** 2018-07-23

**Authors:** Junqing Wang, Yunyun Zhou, Xiaochun Fei, Xuehua Chen, Yongjun Chen

**Affiliations:** 1 Department of Surgery, Ruijin Hospital, Shanghai Jiao Tong University School of Medicine, 197, Rui Jin Er Road, Shanghai, 20025 People’s Republic of China; 2 Shanghai Institute of Digestive Surgery, Ruijin Hospital, Shanghai Jiao Tong University School of Medicine, 197, Rui Jin Er Road, Shanghai, 20025 People’s Republic of China; 3 Shanghai Key Laboratory of Gastric Neoplasms, Ruijin Hospital, Shanghai Jiao Tong University School of Medicine, 197, Rui Jin Er Road, Shanghai, 20025 People’s Republic of China; 4 Department of Data Science, University of Mississippi Medical Center, Jackson, MS 39216 USA; 5 Department of Pathology, Ruijin Hospital, Shanghai Jiao Tong University School of Medicine, 197, Rui Jin Er Road, Shanghai, 20025 People’s Republic of China

## Abstract

Previous studies have reported that the aberrantly expressed AKR1B10 is associated with many cancer development, however the functional roles of AKR1B10 and its regulatory mechanisms in hepatocellular carcinoma (HCC) have been limited studied. In this project, we identified AKR1B10 functional as an oncogene in HCC through tumor/normal human tissue comparison from both GEO microarray and TCGA RNAseq dataset. Further experimental validations from three HCC cell lines (SMMC-7721, HePG2 and HeP3B) also suggested the ontogenetic functions of AKR1B10 in HCC tumor growth. By knocking down AKR1B10 through shRNA in HCC HeP3B cells, we showed it significantly induced cell cycle arrest and inhibited cell growth. Interestingly, integrative analysis of TCGA RNAseq data and miRNA-seq data predicted that miR-383-5p, a novel post-transcriptional tumor suppressor, is negatively associated with AKR1B10 expression. To further investigate the role of miR-383-5p in regulating AKR1B10 in HCC, we performed Dual-luciferase reporter assay experiments. Results showed that miR-383-5p is an upstream modulator targeting AKR1B10 in the post-transcriptional stage. Thus, we report AKR1B10 modulated regulated by miR-383-5p, promotes HCC tumor progress, and could be potentially a therapeutic target for precision medicine in HCC.

## Introduction

World widely, hepatocellular carcinoma (HCC) presents a high incidence rate among human malignancies, ranking a fifth of morbidity and second of malignancy-related mortality^1,2^. Biomarkers aberrantly expressed in HCC at either mRNA or protein stages have been gradually illustrated for HCC tumorigenesis. However, comprehensive and integrative analysis for identifying new therapeutic targets and understanding their functional roles in tumor progression are critically important in the development of novel HCC cancer therapies for precision medicine.

Data mining the public database from Gene Expression Omnibus (GEO) database and the Cancer Genome Altas (TCGA) database provides opportunities for detecting characteristic gene expressions in certain malignancies. In this study, we screened differentially expressed genes (DEGs) by tumor/normal comparison for GEO datasets and explored the significantly enriched Gene Ontology and KEGG pathway in HCC. Then we focused on 3 up-regulated and 11 down-regulated DEGs all shared among in both GEO microarray and TCGA RNAseq data for down streaming analysis. The Search Tool for the Retrieval of Interacting Genes (STRING) database was applied to construct the protein-protein interaction (PPI) network for the shared DEGs and their interacted genes. Among our shared DEGs, Aldo-Keto Reductase Family 1 Member B10 (AKR1B10) was observed as an oncogene participating in the progress of HCC. We explored and verified the expression status of AKR1B10 in both HCC cell lines and tissue samples from our medical center. Knock-down of AKR1B10 in HCC HeP3B cells significantly induced cell cycle arrest and cell growth obstruction.

AKR1B10 is a member of the Aldo/keto reductase super-family, which mainly reduce the aliphatic and aromatic aldehydes efficifently^3^. Accumulating evidence reveals that AKR1B10 functions as a pivotal promotor of human cancers in multiple tissues and organs, including breast, lung, liver, endometrium and gastrointestinal mucosa^3–6^. However, AKR1B10 in HCC has been controversially discussed, and AKR1B10 was described as either a negative prognostic factor or an independent promoting factor of HCC^7–10^. To investigate the AKR1B10 functions, in our study, we performed integrative analysis for TCGA mRNA and miRNA data and built a differentially expressed miRNA-mRNA regulatory network in HCC. Results suggested AKR1B10 is interacted with microRNA-383-5p (miR-383-5p). According to further validation, AKR1B10 was proved to be directly degenerated by miR-383-5p, a tumor suppressor gene in HCC. Herein, we suggest AKR1B10 as a biomarker promoting HCC targeted by miR-383-5p directly. Our findings provide us potential therapeutic targets in metabolism disorder related HCC treatment.

## Results

### Differentially expressed mRNA associated with HCC and pathway analysis

**Figure 1:**
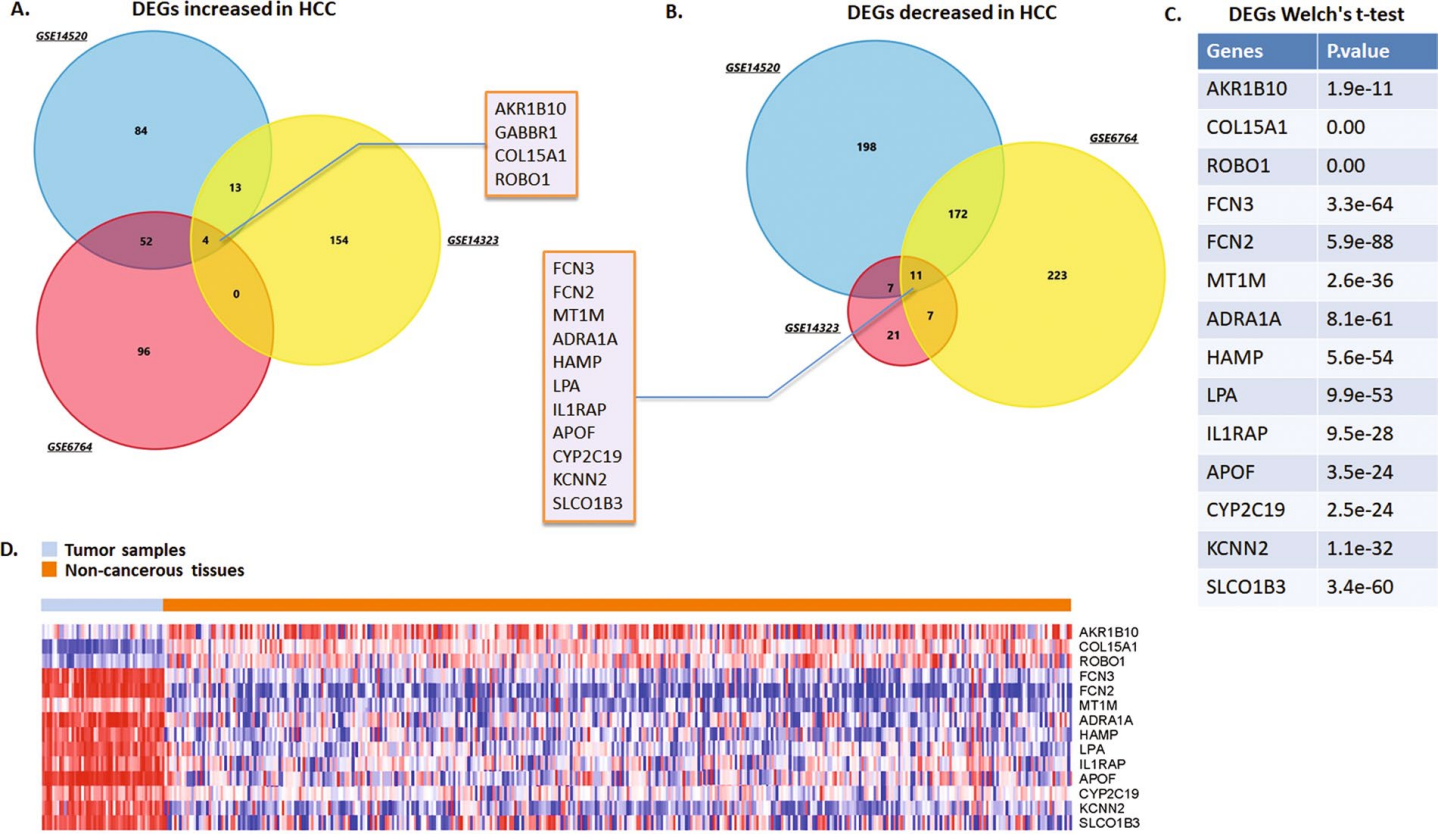
DEGs identified from NCBI GEO datasets (**A**) Venn chart of the over-expressing genes in GSE14520, GSE14323 and GSE6764 liver cancer datasets, which overlapped according to the analysis of FunRich software. Four DEGs extremely over-expressed in the three datasets were selected, including AKR1B10, GABBR1, COL15A1, AKR1B100. (**B**) Venn chart of the decreasing genes in three live cancer datasets. Eleven DEGs extremely decreased were selected, including FCN3, FCN2, MT1M, ADRA1A, HAMP, LPA, IL1RAP, APOF, YP2C19, KCNN2 and SLCO1B3. (**C**) The identified overlapped 15 DEGs from microarray were further tested in TCGA mRNA expression from RNAseq data by Welch’s t-test with *P* < 0.05 as the cutoff for tumor and normal samples comparison. (**D**) Heatmap generated through TCGA database exploration and data mining. Expressions of the 15 DEGs above were presented by hot spots with gradient from red as high expression to blue as low expression.

Based on the criterion of log_2_FC ≥1.5 and *P* value  < 1.0E-04 for selecting DEGs, 403 genes presented amplification in the HCC tissues compared to the non-cancerous samples. Moreover, other 639 genes decreased determined by the same criterion were set. We noticed significant enrichment of the processes closely related to cancer genesis and progress. Results suggested the most significant pathway is the cellular aldehyde metabolic process (*P*-Value: 2.46e-07) (Supp. Table 1). We further focused on 4 over-expressed genes (AKR1B10, GABBR1, COL15A1, ROBO10) out of 403 candidates (Fig. 1A), and 11 down-expressed genes (FCN3, FCN2, MT1M, ADRA1A, HAMP, LPA, IL1RAP, APOF, YP2C19, KCNN2, and SLCO1B3) shared among the three datasets (Fig. 1B). The identified overlapped 15 DEGs from microarray were further tested in TCGA mRNA expression from RNAseq data by Welch’s t-test with *P* < 0.05 as the cutoff for tumor and normal samples comparison. Only the *P* value of GABBR1 gene did not pass the significant cutoff. Therefore we ignored this gene from further analysis (Fig. 1C). The mRNA expression pattern of the 14 DEGs in tumor and normal liver tissues was demonstrated by heatmap in Fig. 1D, through TCGA database exploration of 369 liver hepatocellular carcinoma samples and 49 adjacent specimens.

**Figure 2:**
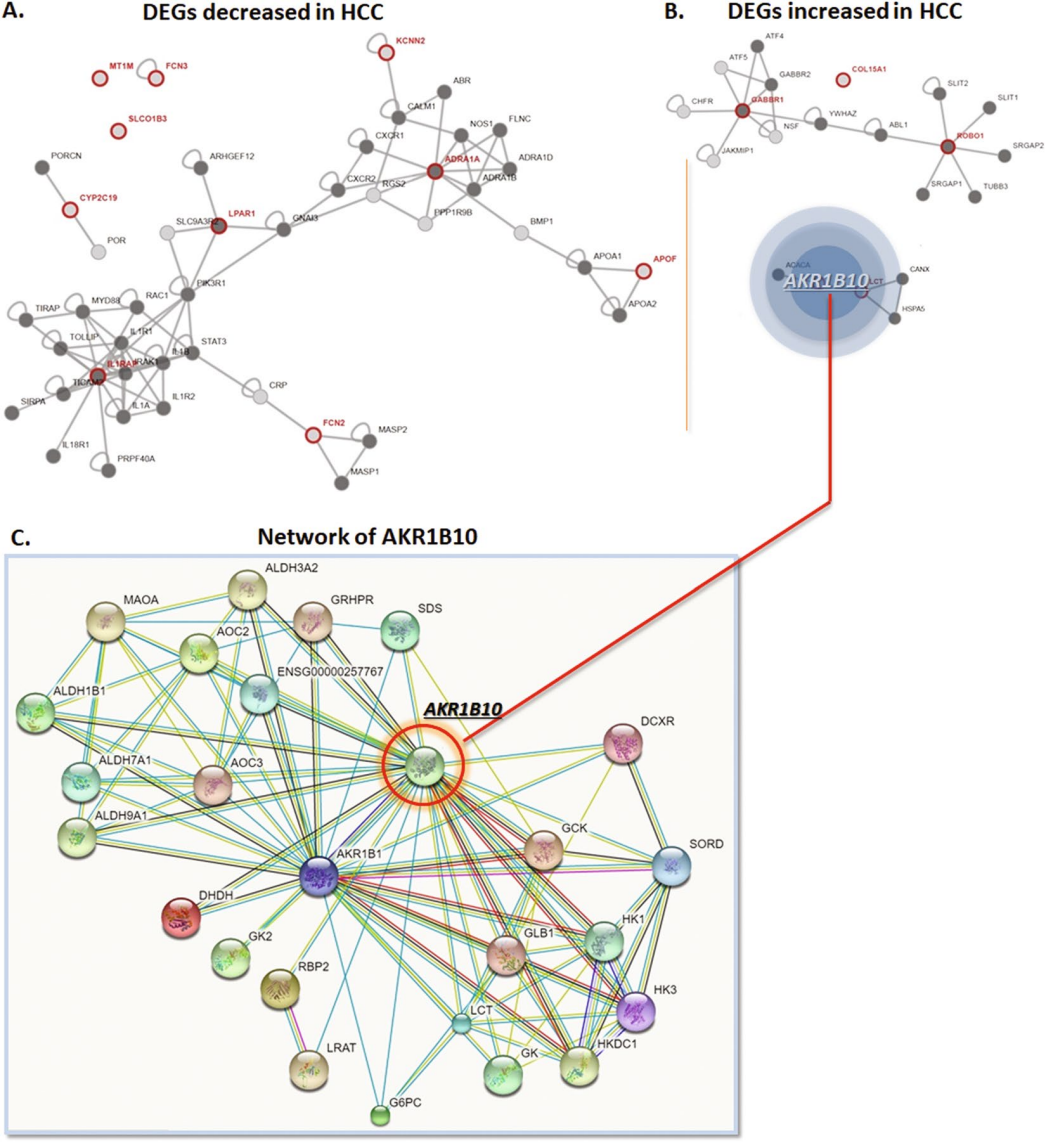
Protein-protein Interaction of the DEGs and selection of AKR1B10. (**A**) Online tool of STRING database analysis of the PPI network for the 11 DEGs decreased in liver cancer datasets. (**B**) Online tool of STRING database analysis of the PPI network for the 4 DEGs over-expressed in liver cancer datasets. (**C**) Amplification of the network for PPI associated with AKR1B10, which demonstrates a complex network between AKR1B10 and proteins critically functions in tumorigenesis and process.

Protein-protein interaction network for the overlapped DEGs from STRING database, and Cytoscape software was shown in Fig. 2A,B. The detailed statistic summaries for the interacted proteins of the DE mRNAs with the combined score cutoff 0.4 predicted by STRING database was shown in Supp. Table 2. Interestingly, AKR1B10, a member of aldo/keto reductase super-family, has been reported as key molecular reducing aliphatic and aromatic aldehydes for cell metabolism^11^. This process is involved in physiological reactions such as the reduction of isoprenyl aldehydes, conversion of retinal to retinol, and bio-transformation of pro-carcinogens^12^. The PPI network for AKR1B10 was shown in Fig. 2C.

### Gene expression pattern of AKR1B10 in the HCC tissues and cell lines

**Figure 3:**
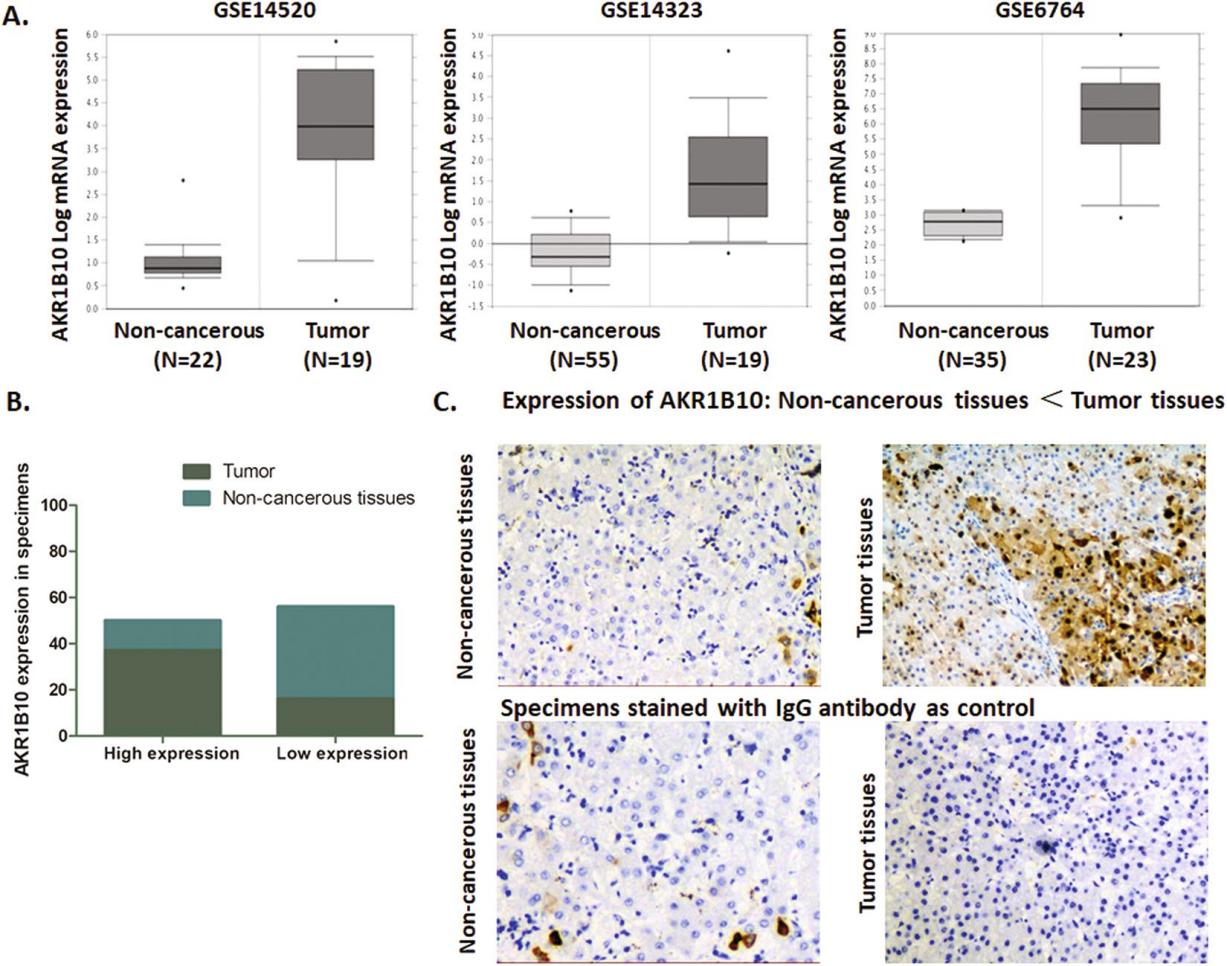
AKR1B10 expression profile in NCBI GEO datasets and HCC patients’ specimens (**A**) Comparison of AKR1B10 mRNA expression status respectively in GSE14520, GSE14323 and GSE6764 liver cancer datasets through *t*-test. AKR1B10 was significantly higher expressed in HCC tissues than that in the non-cancerous tissues (*P* = 3.50E-11 for GSE14323, *P* = 8.17E-7 for GSE14520 and *P* = 6.98E-9 for GSE6764). (**B**) Expression of AKR1B10 was high in 69.81% out of 53 HCC tumor specimens (37/53). On the contrary, most of the non-cancerous tissues presented a relative lower AKR1B10 expression. The expression of AKR1B10 in tumor tissues was frequently and significantly higher than adjacent non-cancerous tissues (*P*  < 0.05). (**C**) Representative graph immunohistochemistry analysis carried out on tissue microarray (200x).

As Fig. 3A showed, the initial exploration of the three databases demonstrated that AKR1B10 presented a significantly higher level of expression in tumor tissues compared to the normal liver tissues (*P* = 3.50E-11 for GSE14323, *P* = 8.17E-7 for GSE14520 and *P* = 6.98E-9 for GSE6764).

To validate the discoveries from the database mining, we explored the expression pattern of AKR1B10 by using IHC assay in our 53 paired HCC tumor and non-cancerous tissue samples. The cases were evaluated according to the expression intensity of AKR1B10, and were separated into low and high AKR1B10 expression group. For the tumor samples, 69.81% (37/53) of the cases were observed highly expressed AKR1B10, and only a portion of 30.19% (16/53) cases presented low AKR1B10 expression. For the non-cancerous specimens paired, highly expressed AKR1B10 was only observed in a portion of 24.53% (13/53) cases, with a 75.47% (40/53) portion presents in low AKR1B10 expression (Fig. 3B,C).

**Figure 4:**
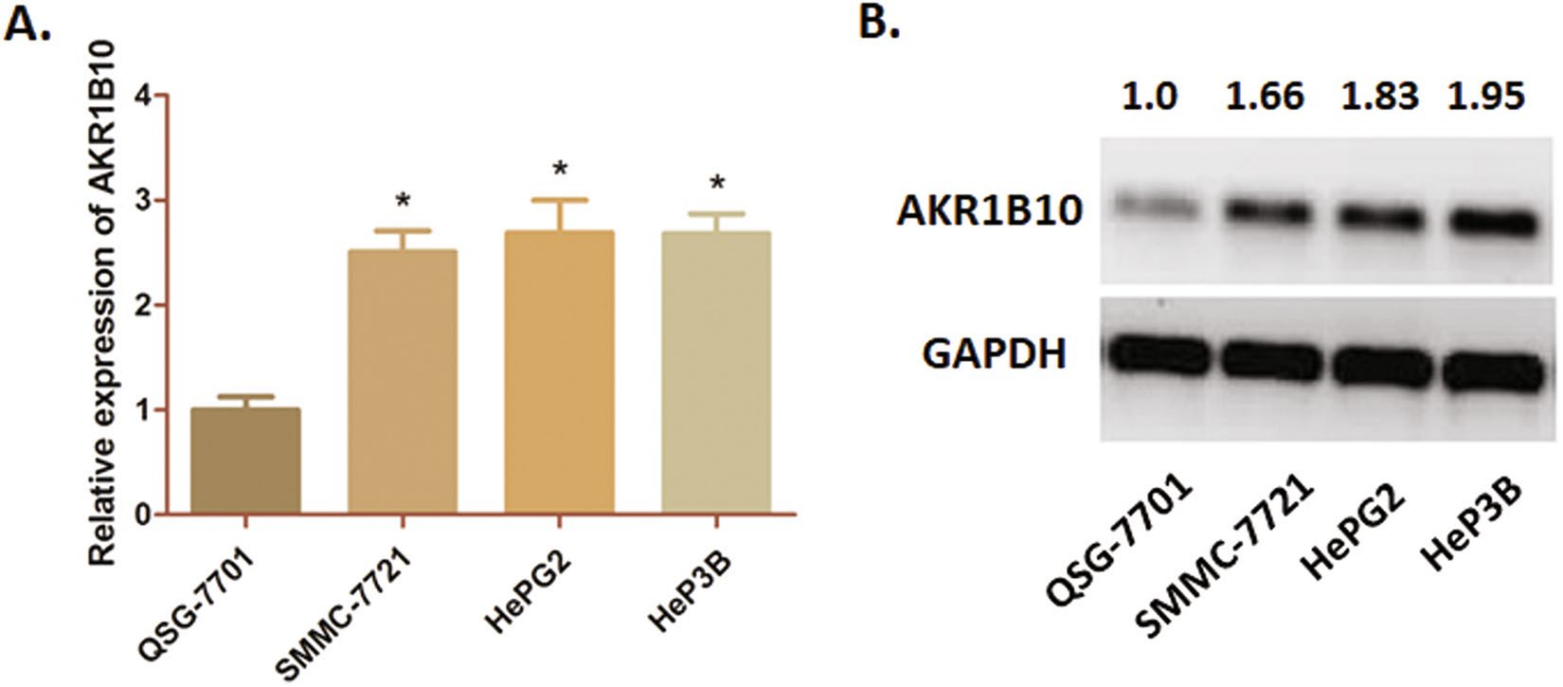
AKR1B10 expression profile in HCC cell lines (**A**) QRT-PCR assay was conducted in three HCC cell lines (SMMC-7721, HeP3B and HePG2) compared with QSG-7701 cells. The mRNA expression of AKR1B10 was significantly higher in HCC cells than that in the control one (*P < 0.05). (**B**) Western blot analysis was carried out, and the blot was cropped and matched according to the columns from the same gel. The protein expression of AKR1B10 was significantly higher in HCC cells than that in the control QSG-7701 cells. Numbers above the blot indicate normalized protein amounts relative to the negative control, as determined by densitometry.

We also observed a similar pattern in three HCC cell lines (SMMC-7721, HeP3B and HePG2), that the mRNA and protein expression of AKR1B10 is significantly increased compared to the control QSG-7701 cells (P < 0.05) (Fig. 4A,B).

### Knock-down of AKR1B10 suppresses cell proliferation and arrests the cell cycle in HeP3B cells

**Figure 5:**
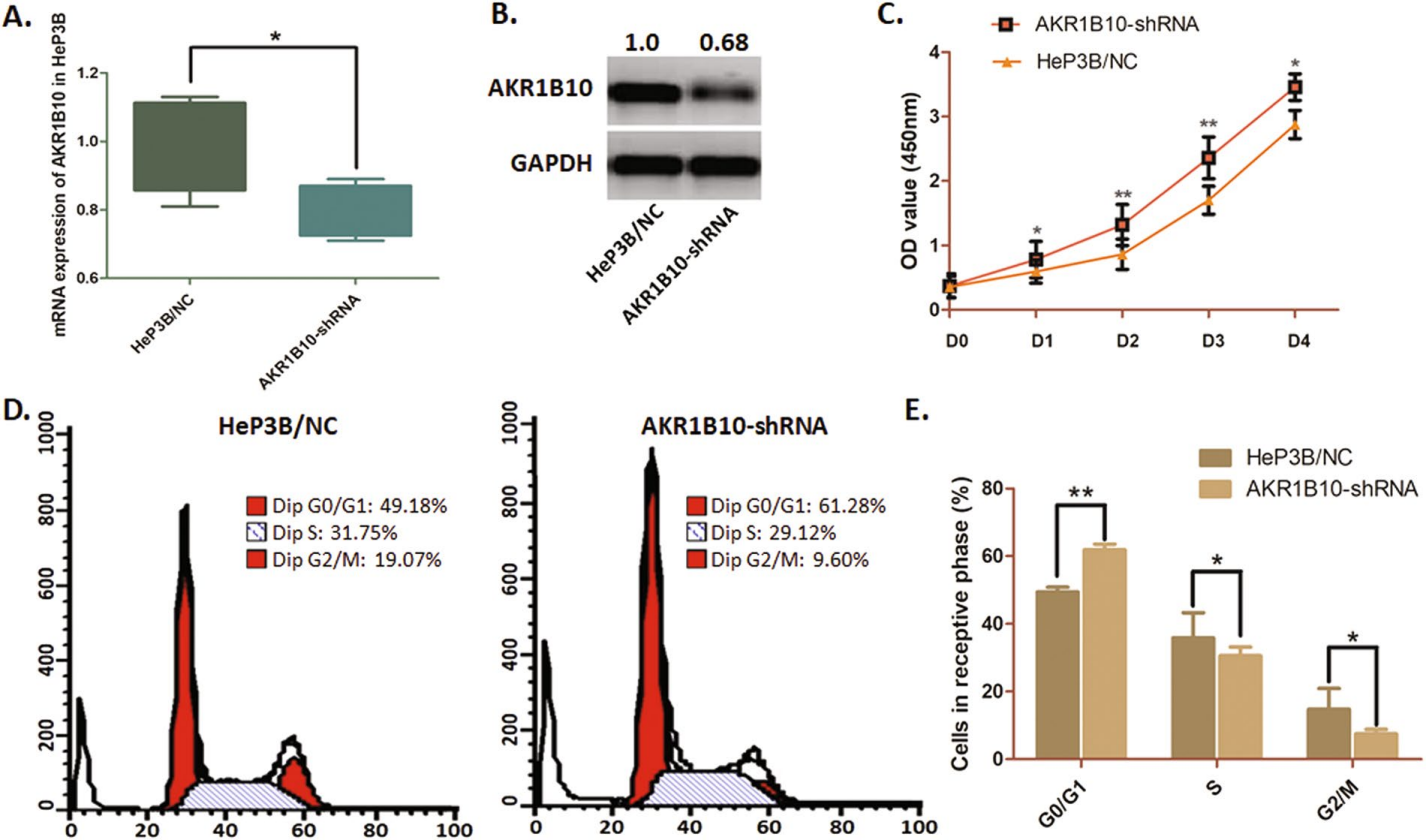
Knock-down AKR1B10 in HeP3B cells impairs cell proliferation and arrests cell cycle. (**A**) QRT-PCR assay verified the significant expression decline of AKR1B10 at mRNA status in HeP3B cells through shRNA transfection by using pGU6/Neo vectors (*P < 0.05). (**B**) Western-blot analysis indicated a significant decrease of AKR1B10 at protein status in HeP3B cells through shRNA transfection by using pGU6/Neo vectors. The blot was cropped and matched according to the columns from the same gel. (**C**) The effect of AKR1B10 on cell proliferation was determined by WST assay. The cell proliferation of HeP3B cells was significantly suppressed when AKR1B10 knocked down (*P < 0.05 for d1 and d4; **P < 0.01 for d2 and d3). (**D**) Representative histograms describing cell cycle profiles of HeP3B cells. The cell cycle was significantly arrested in G0/G1 phase when AKR1B10 knocked down. (**E**) Proportion of cells in various phases of the cell cycle. The results are means of three independent experiments_SD (**P < 0.01, *P < 0.05).

HCC HeP3B cells, presenting a high level of AKR1B10, was selected and transfected with pGU6/Neo vectors to knock down the expression of AKR1B10. Transfection effect was verified through qRT-PCR and Western blot analysis (Fig. 5A,B).

The CCK8 assay demonstrated a significant depression of cell proliferation in AKR1B10 knock down HeP3B cells as *P* value  < 0.05 for d1 and d4, and *P* value  < 0.01 for d2 to d3 (Fig. 5C). Simultaneously, we conducted flow cytometry analysis and found that the cell cycle of HeP3B cells was significantly arrested at G0/G1 phase when AKR1B10 knocked down (Fig. 5D–E). The percentage of HeP3B cells in G0/G1 phase was raised from 49.40% to 61.90% (*P*  < 0.01). The S phase was decreased from 35.82% to 30.59%, and the G2/M phase was decreased from 14.78% to 7.51%. These results indicate that knock-down of AKR1B10 in HeP3B cells significant obstructs the tumor cell growth.

### Differentially expressed miRNA associated with HCC and pathway analysis

As the Heatmap showed in Supp. Figure 2A, we identified 79 differentially expressed miRNAs, 47 down-expressed, and 32 up-expressed miRNAs, from TCGA HCC miRNA-Seq data based on the cutoff threshold (*P* value  < 1.0E-04 and log_2_ FC >= 2). The detailed statistics summary for the 79 DE miRNAs is shown in Supp. Table 3. The functional enrichment analysis for the 79 DE miRNAs was conducted by Funrich Software shown in Supp. Figure 2B–D. Interestingly, consistent with the significant pathway analysis from DE mRNAs, we found the most significant pathway for miRNAs is also related to cellular metabolism (*P*-Value: < 0.001) and transport process (*P*-Value: < 0.001) in a biological process. The results make sense and are consistent with many other scientific findings since liver cancer was believed to be a type of disease due to metabolism disorder. Our results also suggested mRNA and miRNA interact each other in the metabolism process for the HCC development.

### The integrative analysis identified differential miRNA regulatory network in HCC

To evaluate the regulatory relationship between differentially expressed miRNA and mRNA expressions in HCC, we conducted integrative network analysis. The integrative network for DE mRNAs and their interacted genes regulated by down-expressed miRNA and up-expressed miRNAs were shown in Supp. Figure 3A,B respectively. The final prediction of “core” network for cross-talked DE miRNA and mRNA in HCC development was shown in Supp. Figure 4A. We can see that AKR1B10 was predicted to be the target genes of miR-383-5p.

Our results suggested that miRNA deregulation is associated with several steps of cancer initiation and progression. Each miRNA can potentially directly or indirectly regulate the expression of many genes, and a single gene can be targeted by multiple miRNAs. For example, as shown in the Supp. Figure 4A, IL1RAP gene, targeted by several miRNAs, has been reported as an effective therapeutic target in many cancer^13,14^; while miR-130a-5p targeting both ROBO1 and COL15A1, has been reported to be associated with cancer cell migration and implied a useful biomarker in chemotherapy.

The interacted miRNA-mRNA pairs can also be prognostic and predictive biomarkers. As the Kaplan-Meier curve based on HCC survival analysis shown in Supp. Figure 4B–C, high expression of AKR1B10 is associated with poor prognosis, and low expression of miR-383-5p is associated with poor prognosis.

### MiR-383-5p post-transcriptional degenerates AKR1B10 in HeP3B cells

**Figure 6:**
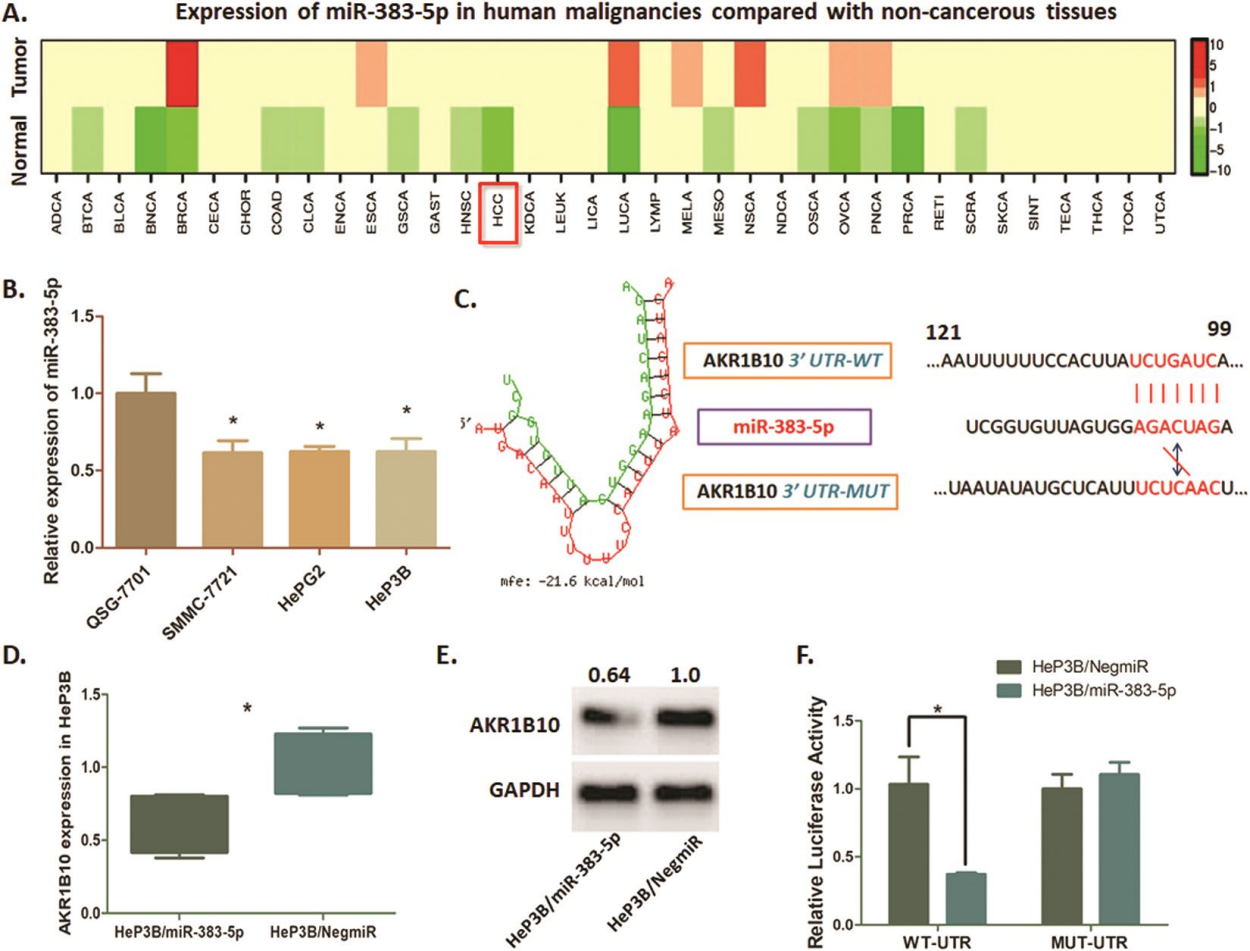
Degeneration of AKR1B10 in HeP3B cells directly regulated by miR-383-5p. (**A**) Expression profile heatmap of miR-383-5p in differential human malignancies generated by dbDEMC (Version 2.0) software. The bar indicates the expression level of objective genes by using color change, from green for low expression to red for high expression. MiR-383-5p is declined in HCC tissues compared with normal liver tissues. (**B**) MiR-383-5p expression detected by qRT-PCR. MiR-383-5p was significantly lower in HCC cells than that in QSG-7701 cells (*P  < 0.05). (**C**) The left graph shows the minimum free energy (MFE) of the combination between miR-383-5p and AKR1B10 mRNA 3′-UTR, and the absolute number of this combination is 21.6 kcal/mol. The right graph is the scheme for the predicted miR-383-5p binding site in the wild type AKR1B10 mRNA 3′UTR (3′UTR-WT), and in the mutant construct (3′UTR-MUT). (**D**) QRT-PCR assay demonstrating the effect of miR-383-5p on AKR1B10 mRNA status in HeP3B cells. AKR1B10 expression was significantly suppressed by over-expressing miR-383-5p in HeP3B cells. (**E**) Western blot analysis shows the degeneration of AKR1B10 expression at protein status in HeP3B cells. AKR1B10 protein was significantly decreased after introducing miR-383-5p into HeP3B cells. The blot was cropped and matched according to the columns from the same gel. (**F**) The direct interaction between miR-383-5p and AKR1B10 was detected by Dual-luciferase reporter assay. Over-expression of miR-383-5p in HeP3B cells (HeP3B/miR-383-5p) significantly decreased the luciferase signal of AKR1B10/pMIR/WT compared with the negative control (HeP3B/NigmiR) (*P  < 0.05). Mutation of the putative miR-383-5p-binding site abolished this suppressive effect.

The expression status of miR-383-5p in differential human malignancies was detected through dbDEMC (Version 2.0) software, and indicated a relatively low expression of miR-383-5p in most of the human tumors including HCC (Fig. 6A). This finding is consistent with our differential expression analysis for miRNAs of TCGA HCC, which further confirmed that miR-383-5p is an inhibitor in HCC cells. As we predicted miR-383-5p as the upstream regulators of AKR1B10 from our network integrative analysis, we performed a qRT-PCR assay to verify our predictions. Results suggested that miR-383-5p in HeP3B cell lines were significantly lower expressed than that in QSG-7701 cells (Fig. 6B).

The potential interaction between miR-383-5p and 3′-UTR of AKR1B10 mRNA was illustrated as Fig. 6C. The Dual-luciferase reporter assay was practiced according to the estimated interacting sequences. The expression of AKR1B10 mRNA and protein appeared to be significantly decreased when miR-383-5p was introduced into HeP3B cells (Fig. 6D,E). Up-regulation of miR-383-5p in HeP3B cells (HeP3B/miR-383-5p) declined luciferase signal, which demonstrated a direct binding and modulation of miR-383-5p on AKR1B10 (Fig. 6F).

## Discussion

As one of the highly aggressive malignancies of human beings, HCC causes suboptimal outcome of overall survive^15^. A large portion of the HCC patients were diagnosed in advanced stages, with no possibility for radical resection or presented poor response to chemotherapy^16^. Currently, molecule-targeted therapy provides us possible therapeutic strategy in anti-cancer treatment. While, as for HCC, there seems no effective molecule-targeted medicine as first-line treatment, except for sorafenib^17,18^. NCBI GEO and TCGA databases, which are containing numerous information of gene expression in multiple human malignancies, provide us the possibility for integrative investigating and seeking for new targets with potential value in cancer treatment.

In this study, we explored the HCC datasets with patients’ information and gene expression profile from NCBI GEO database and TCGA database. DEGs with extremely differential expression profiles in HCC tissues compared with normal liver tissues were set into cohorts for further study. As we observed, 403 genes amplified and 639 ones decreased in HCC specimens were selected from three NCBI GEO datasets (GSE14323, GSE14520 and GSE6764). The results obtained by overlapping these genes provided us 15 genes aberrantly expressed in HCC tissues.

GO and KEGG enrichment pathway relative to the DEGs was detected. A series of pathways involving in tumorigenesis and progress were observed, including cellular aldehyde metabolic process, The oncogenetic functions of AKR1B10 have been reported in many other types of cancer by previous studies. For instance, the inhibition of AKR1B10 significantly suppressed the tumor progress in gastrointestinal cancer, pancreatic cancer, breast cancer etc^19–21^, and over-expressed AKR1B10 induced cisplatin resistance enhancement by positively regulating peroxisome proliferator activated receptor-γ (PPARγ)^6^. Some of the studies also suggested the tumor suppressor effect of AKR1B10 and its clinical relevance^22,23^. Although several studies mentioned the over-expression characteristic of AKR1B10 associated with HCC progression^24–26^, limited studies for the investigations of the regulatory mechanisms for the AKR1B10 interactions with other molecules are continuously needed so as to systematically interpret its functions in HCC development in depth.

In this study, we initially explored differentially expressed genes (DEGs) through patients’ tumor and normal comparison from three NCBI GEO microarray datasets, which are GSE14323, GSE14520, and GSE6764. We explored all the DEGs functional pathways from system biology perspective. Results suggest the top one hit pathway for HCC development is metabolism related. Then, we validated the expression of DEGs in TCGA RNAseq data and focused on the investigation on the 14 DEGs shared in both three GEO microarray datasets and TCGA RNAseq dataset, which contains 3 up-regulated and 11 down-regulated genes. AKR1B10 is identified as one of the top hit over-expressed genes which is associated with metabolism disorder in HCC development.

Further experimental validations of the AKR1B10 expression and functions in either mRNA status or protein status was carried out on both human specimen and cell lines from our medical center. A similar pattern of AKR1B10 expression was observed. IHC assay presented a signigicantly higher expression of AKR1B10 in HCC tumor tissues compared to the adjacent non-cancerous tissues. Three HCC cell lines we detected also showed higher AKR1B10 than the control QSG-7701 cells. Then we explored the functions of AKR1B10 in HCC tumorigenesis process by knocking down the AKR1B10 through shRNA in HCC HeP3B cells. As we observed, the mRNA and protein expression of AKR1B10 was significantly declined after transfection. Along with the expression impairing of AKR1B10, the cell growth of HeP3B cells presented to be meeting with a remarkable stagnation, and the cell cycle was observably arrested in G0/G1 phase. The findings above indicate that AKR1B10 functions to be a promotor inducing HeP3B cell growth in tumor process.

The molecular mechanism conferring to AKR1B10 amplification is valuable for our intensive understanding on HCC progress. Knowledgeably, post-transcriptional regulation is a pivotal approach leading to tumor promotion or suppression^27^. To elucidate the regulatory functions of AKR1B10, we performed integrative network analysis from differentially expressed miRNA and mRNAs from TCGA HCC RNAseq data. We used the interactions data from miRWalk2.0 database to generate a global miRNAs and their target genes network. According to this analysis, we discovered miR-383-5p is the upstream molecular and interacted with AKR1B10 which was highly related to the metabolism pathway the as one of the most critical factors in the development of HCC by GO enrichment analysis and pathway analysis.

The miR-383-5p has been reported as a tumor suppressor in various human cancer including HCC^28–31^. For example, miR-383-5p inhibited tumor cell mobility through regulating EPAS1 in lung cancer cells^28–31^, the decrease of miR-383-5p is suggested as a potential independent biomarker for poor prognosis in intestinal subtype of gastric cancer^26^, and the rescue of miR-383-5p expression in HCC cells prompted a better response outcome by the sorafenib targeting therapy^32^. Our results from bioinformatics analysis and experimental validations in this project are consistent with their findings.

Our qRT-PCR assay demonstrated that miR-383-5p expression was significantly decreased in HCC HeP3B cell line. The interactions of miR-383-5p and AKR1B10 were tested by the Dual-luciferase reporter assay. The post-transcriptional regulatory relationship between miR-383-5p and 3′-UTR of AKR1B10 mRNA was evidenced by the decreased luciferase signal by building their direct interaction. Logically, degeneration of AKR1B10 in HCC cells could partly explain the tumor inhibiting mechanism that miR-383-5p induced in tumors. The survival analysis suggested the inverse association of expression alteration for miR-383-5p and AKR1B10 with survival outcome. This clinical evidence again confirmed the negative correlation between miR-383-5p and AKR1B10. Even, for the targeting therapy with sorafenib, the rescue of miR-383-5p expression in HCC cells prompts an approving outcome.

As summarized, integrative bioinformatics analysis through NCBI GEO database and TCGA database identified AKR1B10 as an oncogene interacted with miR-383-5p and suggested their involvement of metabolism pathway in HCC development. *In vitro* experiment combining with clinical specimen study verified the high expression of AKR1B10 in HCC specimens and cell lines. Knock-down AKR1B10 significantly declined HeP3B cells’ ability of growth, which indicated AKR1B10 as a promotor in HCC process. Tumor suppressing miR-383-5p was verified as the up-stream regulator modulating AKR1B10 expression in the process of HCC development.

All these information suggested that AKR1B10 might be one of the pivotal molecules involved in HCC tumorigenesis and progress. More experimental validations to investigate other predicted miRNA regulatory functions can be performed in the future.

## Methods and Materials

### Statements

1. The experimental protocols were approved by Ruijin Hospital, Shanghai Jiao Tong University School of Medicine.
2. The methods applied in this study described below were carried out in accordance with relevant guidelines and regulations.
3. We declare that the informed consent was obtained from all the subjects. For any subject under 18 years old, the consent was obtained from a parent and/or the legal guardian.

### Data resource and Description

The overall workflow of this study design is summarized as shown in Supp. Figure 1. Three expression microarray datasets GSE14323, GSE14520 and GSE6764 containing HCC tumor and normal samples were downloaded from the NCBI GEO dataset (https://www.ncbi.nlm.nih.gov/geo/). Platforms of GEO datasets for GSE14323 and GSE14520 dataset are GPL3921 (Affymetrix HT Human Genome U133A Array), and for GSE6764 is GPL570 (Affymetrix Human Genome U133 Plus 2.0 Array) (Agilent Technologies, Santa Clara, CA, USA). The GSE14323 dataset contains 74 samples with 55 HCC tumor and 19 normal samples; The GSE14520 dataset contains 44 samples with 22 tumor and 19 normal samples; and, the GSE6764 dataset contains 58 samples with 35 tumor and 23 normal samples.

TCGA liver hepatocellular carcinoma mRNA and miRNA mature strand RSEM normalized counts data derived from RNAseq Illumina Hiseq platform were downloaded from UCSC Xena database (https://xenabrowser.net/). TCGA RNAseq data contains 418 samples with 369 primary tumor and 49 solid tissue normal samples. The miRNA mature ID was annotated by R miRBaseConverter package^33^.

### Bioinformatics analysis for identifying differentially expressed mRNAs in HCC microarray and their functional analysis

Raw .CEL files of the microarray from each GEO dataset were normalized by quantile method of Robust Multichip Analysis (RMA) from R affy package^34^. Significant analysis for DEGs was performed by tumor and normal comparison from R samr package^35,36^. The cutoff value for DEGs selection in each dataset is based on the following criteria: log_2_ FC ≥1.5 and *P* value  < 1.0E-04.

A combined DEGs list from all the three datasets, which is comprised of 403 upregulated and 639 down-regulated genes, were identified as a union. Gene Ontology and KEGG functional pathway enrichment were conducted by Database for Annotation Visualization and Integrated Discovery tools (DAVID).

We used Venn Gram to show the 4 up-regulated and 11 down-regulated genes shared among the three microarray datasets. The results of Protein-Protein Interaction analysis (PPI) for 15 DE mRNAs were performed by STRING database (http://string-db.org).

### Surgical specimens and cell culture

Fifty-three HCC specimens from patients performed partial hepatectomy without preoperative therapy were detected by using immunohistochemistry analysis in our medical center during 2015–2016, paired with adjacent non-cancerous tissues.

Three HCC cell lines (SMMC-7721, HePG2 and HeP3B) were purchased from Shanghai Institutes for Biological Sciences, Chinese Academy of Science (Shanghai, China), along with the normal human hepatic cell line QSG-7701. Hep3B cells, which mimick miR-383-5p high-expression were constructed as we previously described for Dual-luciferase report assay, and the control ones were set^37,38^. Cells culture medium were set, at 37 °C with an atmosphere of 5% CO_2_ in the humidified cell incubator, as the RPMI 1640, which was supplemented with 10% heat-inactivated fetal bovine serum (FBS), 100 ug/ml streptomycin and 100U/ml Penicillin.

### Immunohistochemistry analysis and Western blot analysis

Antibodies against AKR1B10 were introduced (Abcam, USA) following the customer instruction. IgG antibody was utilized as control in IHC assay. Two professional pathologists were assigned for inspecting the tissues blindly. RIPA buffer containing Protease Inhibitor Cocktail (Pierce, USA) was used to lyse the cells, and the protein concentration was measured by BCA Protein Assay Kit (Pierce, USA). Proteins were electrophoresed and electrotransfered. Antibody against AKR1B10 (1:1000) and GAPDH (1:5000) were probed. Horseradish peroxidase-conjugated secondary antibody was used for further probe. Protein quantity was detected by using GAPDH as a loading control.

### RNA isolation and Real-time qPCR assay

TRIzol reagent (Invitrogen, USA) was used for total RNA was extracted from cell lines. The first-strand cDNA was synthesized using High-Capacity cDNA Reverse Transcription Kit (ABI, USA). RT-primers of AKR1B10 mRNAs were synthesized as follows: 5′-GACCCCTTGTGAGGAAAGCC-3′ (forward) and 5′-ATTGCAACACGTTACAGGCCC-3′ (reverse) (Sangon Biotech Company, Shanghai, China). TaqMan Gene Expression Assays was utilized for Real-time quantitative polymerase chain reaction (qRT-PCR) (ABI, USA).

### Cell transfection

HeP3B cells were transfected by pGU6/Neo vectors (GenePharma, Shanghai, China) containing shRNA suppressing AKR1B10 translation or non-containing ones. We cultured and selected the cells in medium containing 400 μg/ml G418 (Santa Cruz, USA). Stably transfected cells above were validated by qRT-PCR and Western blot analysis compared with the negative control cells. All cells were cultured and maintained in medium containing 200 μg/ml G418. MiR-383-5p was introduced into HeP3B cells through the mimic method for further Dual-luciferase Reporter Assay.

### Cell proliferation assay and cell cycle analysis

1 × 10^**6**^ HeP3B cells stable transfected or the negative control cells were cultured in 96-well microtiter plates in triplicate and incubated for 5 days at 37 °C with an atmosphere of 5% CO_2_. OD was measured by using microplate computer software (Bio-Rad Laboratories, USA) according to the protocol of CCK8 Assay Kit (Dojindo, Japan). The curves of cell proliferation were plotted.

Cells above were treated by ethanol fixation, RNase A treatment, and Propidium Iodide staining, and then were detected under flow cytometry by FACSCalibur (Becton Dickinson, USA). Cell populations at the G0/G1, S and G2/M phases were quantified by Modfit software (Becton Dickinson, USA) excluding a calculation of cell debris and fixation artifacts.

### DEGs analysis for TCGA mRNA and miRNA expression and the functional analysis

The identified overlapped 15 DEGs from microarray were further tested in TCGA mRNA expression from RNAseq data by Welch’s t-test with *P* < 0.05 as the cutoff for tumor and normal samples comparison. Only the *P* value of GABBR1 gene did not pass the significant cutoff. Therefore we removed this gene from further analysis.

2172 mature miRNAs strand expression profiling was carried out from TCGA tumor and normal comparison. After filtering miRNAs that were not expressed in most of the samples, 1270 miRNAs were used for further differential expression analyses by R samr package^35,36^ with default settings (*P* < 1.0E-04). Any miRNAs with less than 2 Fold change were further removed from the downstream analysis.

### The miRNA –mRNA network integrative analysis

To further investigate the DE miRNAs regulatory mechanism in HCC, we generated an interaction network by integrating TCGA HCC DE miRNA-mRNA co-expression network and miRNA-target genes (TG) network based on database searching. For the construction of the TCGA miRNA-mRNA co-expression network, we built the affinity matrix based on the similarity cutoff of miRNA-mRNA pairs connectivity. We set the cutoff for selecting the significant miRNA and their co-expressed mRNA pairs based on the following criteria: Pearson correlation *P*-Value less than *P* < 1.0E-04. For building the miRNA-target genes (TG) epigenetic network, we mapped our TCGA DE miRNA to the miRWalk2.0 database, which curated the connectivity of miRNA with their targeted genes from 12 existing miRNA database such as MirTarbase, miRanda and TargetScan, to identify target genes of DE miRNAs from the TCGA HCC patient data. Totally 10753 genes mapping to the target genes of 79 DE miRNA were identified. Then we merged the co-expression DE miRNA-mRNA network with the miRNA-TG network by an iGraph tool from R package. Finally, the “core” network was refined using R package SpidermiR tool by only keeping the target genes overlapped with 14 DE mRNAs and their interacted genes predicted by STRING database with the cutoff value of combined score more than 0.4. The final HCC-specific miRNA-mRNA regulatory network was constructed by merging the co-expression weighted network derived from DEGs of TCGA HCC dataset and the miRNA-target genes (TG) network derived from database searching.

### Experimental validation for Direct interaction between AKR1B10 mRNA and miR-383-5p

According to the analysis, microRNA-383-5p (miR-383-5p) appears directly binding to the 3′ UTR of AKR1B10 mRNA. Dual-luciferase report assay was conducted to verify the exact binding between miR-383-5p and AKR1B10. A 198 bp sequence from the 3′-UTR of AKR1B10 mRNA containing the potential miR-383-5p binding site was set as follow: 5′-aggtgctgttttagacatttatttctgtatgttcaactaggatcagaatatcacagaaaagcatggcttgaataaggaaatgacaattttttccacttatctgatcagaacaaatgtttattaagcatcagaaactctgccaacactgaggatgtaaagatcaataaaaaaaataataatcataaccaacaaaaaaaaa-3′. The correspondingmutant sequence were constructed by Sangon Biotech Company (Shanghai, China) as follow: 5′-gtgctccagatattgtcttatttatgtctttctactagttgcaactgtaaaactctgtataccttcggtagtaaatgcataagtctaatatatgctcatttgtcaactgtagataagataaattaccttgacatagtgtccgatctcagtgcaagaatacaactaaatatatatttaaattgaaatcgatctatatatt-3′. The above sequences were respectively cloned into pMIR-REPORT luciferase vectors (Promega, USA), which contains Firefly luciferase. Moreover, the pRL-TK vectors, which contains Renilla luciferase was applied as a control. Hep3B cells transfected with miR-383-5p mimics or the negative control were co-transfected with the vectors. Luciferase activity was measured by using Dual-Glo Luciferase assay system (Promega, USA) 48 h post-transfection.

### Survival analysis and statistics analysis for the experiment data

Survival analysis of AKR1B10 was evaluated through the application of the kmplot tool (http://www.kmplot.com) including 364 liver cancer patients with overall survival information. The survival analysis for miR-383-5p expression is from 614 liver cancer patients curated by kmplot with default settings. For the expression of both AKR1B10 and miR-383-5p, the best performing threshold was used for the Univariate Cox regression analysis after screening the lower and upper expression quartiles. The hazard ratio with 95% confidence and *P*-Value from the log-rank test were calculated by kmplot tool.

The experimental data were statistically analyzed by using SPSS 18.0. Fisher’s exact test was applied to categorical outcomes; AVONA and t-test were performed on continuous outcomes from the experiments for *P* values calculation. A *P* value < 0.05 was considered as significant statistically.

## Electronic supplementary material

Supplementary Material (Main)

Supplementary Table 2

Supplementary Table 3
