# Supplementary material for: Biostatistics mining associated method identifies AKR1B10 enhancing hepatocellular carcinoma cell growth and degenerated by miR-383-5p
Source: Sci Rep. 2018 Jul 23;8:11094. doi: 10.1038/s41598-018-29271-3 (PMC6056456; doi:10.1038/s41598-018-29271-3)
Supplement: Supplementary file 1 — Supplementary Material (Main) [file 41598_2018_29271_MOESM1_ESM.pdf]

Supplementary information

Biostatistics mining associated method identifies AKR1B10 enhancing hepatocellular carcinoma cell growth and degenerated by miR-383-5p

Junqing Wang [a,b,c,1] *, Yunyun Zhou [d,1] , Xiaochun Fei [e], Xuehua Chen [b,c], Yongjun Chen [a] *

[a] Department of Surgery, Ruijin Hospital, Shanghai Jiao Tong University School of Medicine, 197, Rui Jin Er Road, Shanghai 20025, People's Republic of China

[b] Shanghai Institute of Digestive Surgery, Ruijin Hospital, Shanghai Jiao Tong University School of Medicine, 197, Rui Jin Er Road, Shanghai 20025, People's Republic of China

[c] Shanghai Key Laboratory of Gastric Neoplasms, Ruijin Hospital, Shanghai Jiao Tong University School of Medicine, 197, Rui Jin Er Road, Shanghai 20025, People's Republic of China

[d] Department of Data Science, University of Mississippi Medical Center, Jackson, MS 39216, USA

[e] Department of Pathology, Ruijin Hospital, Shanghai Jiao Tong University School of Medicine, 197, Rui Jin Er Road, Shanghai 20025, People's Republic of China

[1] Both authors contributed equally to this study.

* Corresponding author at: Department of Surgery, Ruijin Hospital, Shanghai Jiao Tong University, School of Medicine, 197, Rui Jin Er Road, Shanghai 20025, People's Republic of China.

E-mail addresses: wangjunqingmd@hotmail.com

Supp. Table. 1: Significant enrichment of the processes closely related to HCC cancer genesis and progress

Category	ID	Term	*P*-value
BP	GO:0006081	Cellular aldehyde metabolic process	2.46E-07
BP	GO:0055114	Oxidation-reduction process	2.46E-07
BP	GO:0022617	Extracellular matrix disassembly	3.04E-08
BP	GO:0007155	Cell adhesion	3.31E-04
BP	GO:0000904	Cell morphogenesis involved in differentiation	5.86E-06
BP	GO:0048667	Cell morphogenesis involved in neuron differentiation	3.56E-08
BP	GO:0010517	Regulation of phospholipase activity	1.10E-08
BP	GO:0044281	Small molecule metabolic process	1.96E-08
BP	GO:0002252	Immune effector process	9.63E-07
BP	GO:0034097	Response to cytokine	9.09E-09
BP	GO:0019221	Cytokine-mediated signaling pathway	9.46E-09
BP	GO:0034142	Toll-like receptor 4 signaling pathway	1.34E-06
BP	GO:0007166	Cell surface receptor signaling pathway	1.08E-05
BP	GO:0051049	Regulation of transport	2.04E-07
KEGG pathway	hsa01100	Metabolic pathways	1.25E-10
KEGG pathway	hsa04512	ECM-receptor interaction	2.80E-10
KEGG pathway	hsa04151	PI3K-Akt signaling pathway	5.81E-07
KEGG pathway	hsa05204	Chemical carcinogenesis	6.58E-16
KEGG pathway	has04210	Apoptosis	1.42E-12
KEGG pathway	Has04064	NF-kappa B signaling pathway	3.41E-08
KEGG pathway	Has04620	Toll-like receptor signaling pathway	5.73E-08
KEGG pathway	Has04010	MAPK signaling pathway	3.71E-06

Supp. Table. 2: The detailed statistic summaries for the interacted proteins of the DE mRNAs with the combined score cutoff 0.4 predicted by STRING database
(Shown as 'Supplementary Dataset' in the form of an Excel document named 'Supp. Table.2')

Supp. Table. 3: The detailed statistics summary for the 79 DE miRNAs
(Shown as 'Supplementary Dataset' in the form of an Excel document named 'Supp. Table.3')

Supplementary Figures and legends

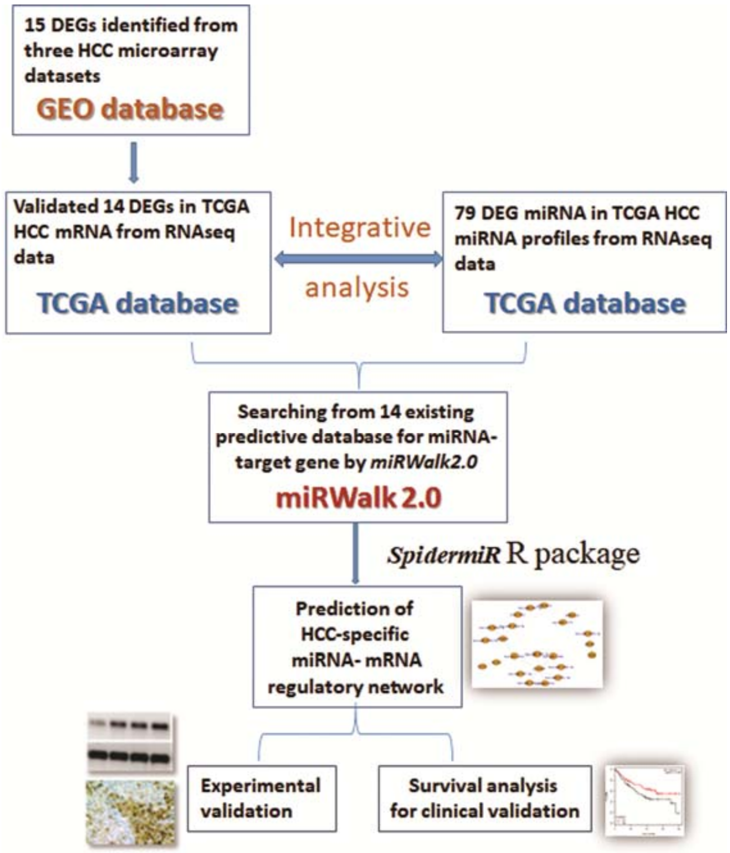

Supp. Fig. 1. The overall workflow in this study

The Figure demonstrates the overall project design of this study.

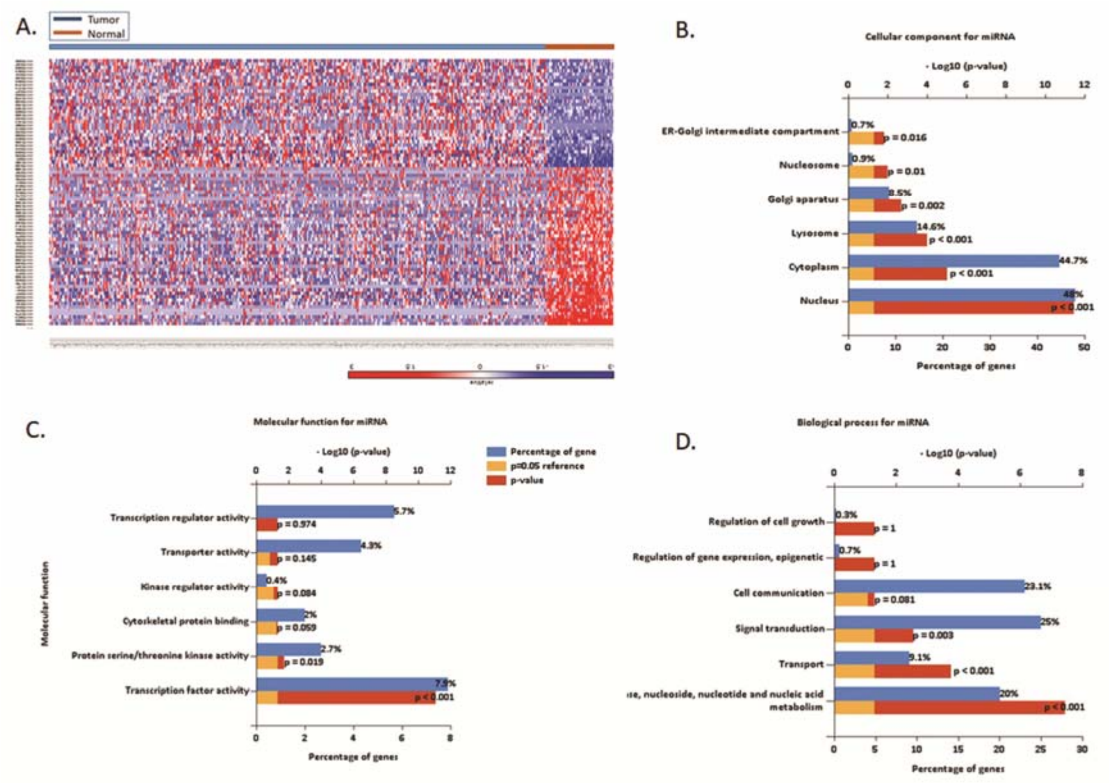

Supp. Fig. 2. The DE miRNA functional enrichment analysis in HCC

(A) Heatmap of 79 differentially expressed miRNA by tumor and normal comparison from TCGA HCC miRNA-seq data. Heatmap generated through TCGA datasets analysis. Among the 79 DE miRNAs, 47 miRNAs were low expression in HCC and 32 miRNAs were over expression in HCC. (B)～(D) Gene Ontology(GO) Functional enrichment analysis shows the top hit GO terms including Biological process, Molecular functions, and Cellular compenent for the 79 DE miRNAs performed by Funrich Software.

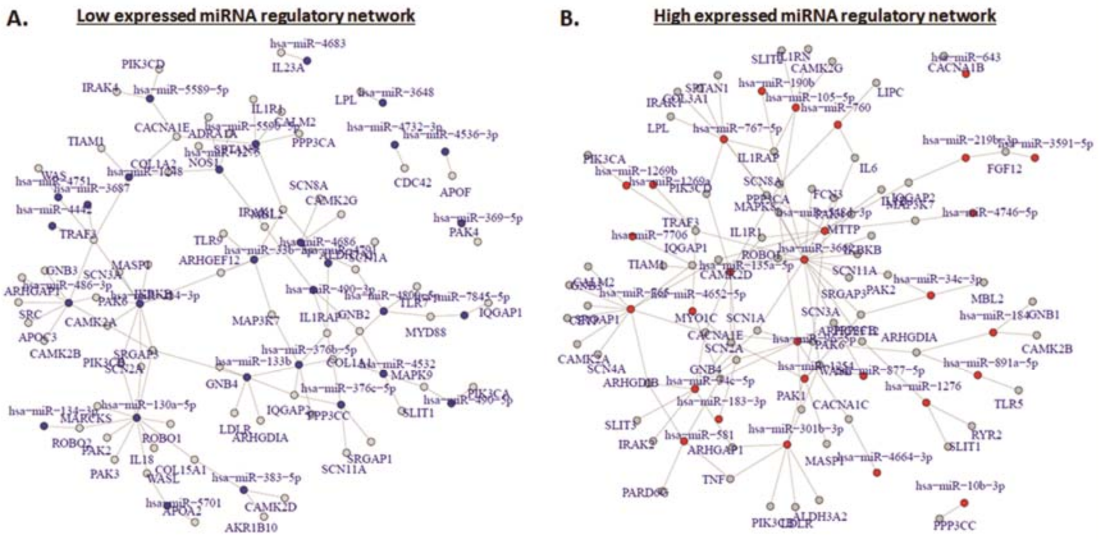

Supp. Fig. 3. The DE miRNA target genes interaction network

(A) Low expressed miRNA regulatory network for HCC-specific target genes; (B) High expressed miRNA regulatory network for HCC-specific target genes.

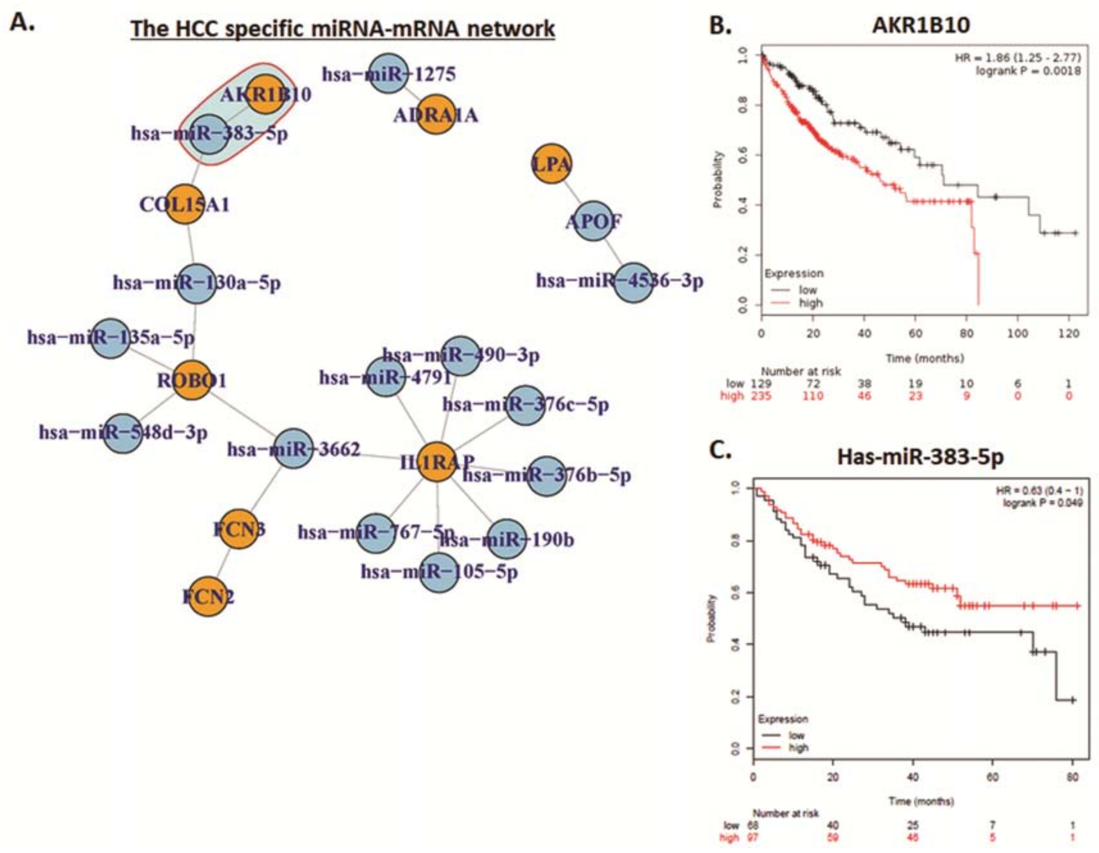

Supp. Fig. 4. HCC-specific miRNA-mRNA network establishment and the

survival analysis for AKR1B10 and miR-383-5p in HCC

(A) The HCC specific miRNA-mRNA network from network integrative analysis. The relationship between AKR1B10 and miR-383-5p is highlighted. (B) Kaplan-Meier plotter for survival analysis of AKR1B10 in HCC patients. (C) Kaplan-Meier plotter for survival analysis of miR-383-5p in HCC patients.
